# Safety and Immunogenicity of a Heterologous Prime-Boost Ebola Virus Vaccine Regimen in Healthy Adults in the United Kingdom and Senegal

**DOI:** 10.1093/infdis/jiy639

**Published:** 2018-11-08

**Authors:** Navin Venkatraman, Birahim Pierre Ndiaye, Georgina Bowyer, Djibril Wade, Saranya Sridhar, Daniel Wright, Jonathan Powlson, Ibrahima Ndiaye, Siry Dièye, Craig Thompson, Momar Bakhoum, Richard Morter, Stefania Capone, Mariarosaria Del Sorbo, Sophie Jamieson, Tommy Rampling, Mehreen Datoo, Rachel Roberts, Ian Poulton, Oliver Griffiths, W Ripley Ballou, François Roman, David J M Lewis, Alison Lawrie, Egeruan Imoukhuede, Sarah C Gilbert, Tandakha N Dieye, Katie J Ewer, Souleymane Mboup, Adrian V S Hill

**Affiliations:** 1 Jenner Institute, University of Oxford, United Kingdom; 2 Centre Hospitalier Universitaire le Dantec, Dakar, Senegal; 3 ReiThera Srl, Rome, Italy; 4 GlaxoSmithKline Biologicals, Rixensart, Belgium; 5 National Institute for Health Research/Imperial Clinical Research Facility, Hammersmith Hospital, London, United Kingdom

**Keywords:** Ebola, vaccine, viral vectors, ChAd3, MVA; EBO-Z

## Abstract

**Background:**

The 2014 West African outbreak of Ebola virus disease highlighted the urgent need to develop an effective Ebola vaccine.

**Methods:**

We undertook 2 phase 1 studies assessing safety and immunogenicity of the viral vector modified vaccinia Ankara virus vectored Ebola Zaire vaccine (MVA-EBO-Z), manufactured rapidly on a new duck cell line either alone or in a heterologous prime-boost regimen with recombinant chimpanzee adenovirus type 3 vectored Ebola Zaire vaccine (ChAd3-EBO-Z) followed by MVA-EBO-Z. Adult volunteers in the United Kingdom (n = 38) and Senegal (n = 40) were vaccinated and an accelerated 1-week prime-boost regimen was assessed in Senegal. Safety was assessed by active and passive collection of local and systemic adverse events.

**Results:**

The standard and accelerated heterologous prime-boost regimens were well-tolerated and elicited potent cellular and humoral immunogenicity in the United Kingdom and Senegal, but vaccine-induced antibody responses were significantly lower in Senegal. Cellular immune responses measured by flow cytometry were significantly greater in African vaccinees receiving ChAd3 and MVA vaccines in the same rather than the contralateral limb.

**Conclusions:**

MVA biomanufactured on an immortalized duck cell line shows potential for very large-scale manufacturing with lower cost of goods. This first trial of MVA-EBO-Z in humans encourages further testing in phase 2 studies, with the 1-week prime-boost interval regimen appearing to be particularly suitable for outbreak control.

**Clinical Trials Registration:**

NCT02451891; NCT02485912.

## Teaser

New vaccines are needed for outbreak pathogens and novel technologies to manufacture them. We describe 2 phase 1 clinical trials demonstrating safety and immunogenicity of a novel Ebola vaccine manufactured using an improved method to reduce costs and increase yield.

The largest recorded outbreak of Ebola virus disease (EVD) resulted in >11300 deaths in West Africa and highlighted the urgent need for development of an efficacious vaccine [1]. This led to the accelerated development of potential vaccine candidates that can be used both in an outbreak setting and to provide long-term protection in populations at risk of sporadic outbreaks [2]. A number of vaccines have been evaluated in phase 1 trials including DNA vaccines, virus-like particles, and viral vectors such as live replicating vesicular stomatitis virus (rVSV), human and chimpanzee adenoviruses, and recombinant modified vaccinia virus [3–11]. The landmark ring vaccination trial in Guinea provided the first evidence of an Ebola vaccine, rVSV expressing the Zaire Ebolavirus (ZEBOV) glycoprotein, which is highly efficacious and could be stockpiled to curtail future outbreaks [4, 12]. Whether this single-dose vaccine provides durable protection is yet to be elucidated. In addition, it targets only 1 Ebola species and whether it will be licensed remains unclear [13].

The use of different viral vectors, namely a replication-deficient chimpanzee adenovirus followed by a modified vaccinia Ankara (MVA) virus encoding the same glycoprotein (GP) in a heterologous prime-boost regimen is a leading strategy for developing vaccine regimens with higher potency, immediate protection, and better durability. Simian adenovirus vectors are an attractive vaccine platform as the viruses from which they are derived are not known to cause infections in humans and there is consequently a low human seroprevalence of antibodies to the chimpanzee adenovirus 3 (ChAd3) used here and to other simian vectors [14]. ChAd3 has been previously tested as a potential vaccine candidate for other infectious diseases, including hepatitis C and human immunodeficiency virus [15, 16]. It was also recently evaluated as a vector for potential Ebola vaccines in a number of clinical trials in the United Kingdom, Europe, and Africa [6, 7, 10, 17]. Overall, these studies have demonstrated the safety of the recombinant ChAd3 vectored Ebola Zaire (ChAd3-EBO-Z) vaccine and as a nonreplicating vector, it has not caused any adverse events (AEs) of significant concern (arthralgia and significant rates of postvaccination fever) such as those reported after vaccination with the replication-competent rVSV vector [3].

The induction of both antibodies and CD8^+^ T-cell responses is potentially protective against EVD [18, 19]. ChAd3-EBO-Z administered alone has been shown to induce both antibody and T-cell responses in humans [7]. Although rVSV-ZEBOV elicits comparable humoral responses, there is less evidence of durable cellular immunogenicity induced by this vaccine, particularly with lower doses [3, 8, 20]; an ongoing phase 2 trial of these vaccines in Liberia (Partnership for Research on Ebola Vaccines in Liberia [PREVAIL]) will directly compare both the nature and durability of immunity [21, 22]. In macaques, the administration of a ChAd3-vectored vaccine required a boost with an MVA-vectored vaccine to generate a durable protective response to lethal Ebola virus (EBOV) challenge [18]. Recent human studies in the United Kingdom and Africa have shown that boosting with an MVA-vectored vaccine encoding multiple filovirus genes resulted in several-fold higher antibody and T-cell responses, which remained higher at 6 months after boost, compared to 6 months after administration of ChAd3-EBO-Z alone [7, 10]. A very short prime-boost interval would be of great value in an outbreak setting for use in frontline workers or in a ring vaccination strategy. Alternatively, a longer interval might confer durable protection, and be of use in populations at risk of sporadic outbreaks.

Here we evaluate a new Ebola vaccine candidate, MVA-EBO-Z (modified vaccinia Ankara virus vectored Ebola Zaire vaccine), manufactured on an immortalized duck retinal cell line, instead of primary chick embryo fibroblast cells, which have been used to manufacture all previous MVA-vectored vaccines tested in clinical trials to date. We also compared a 1-week prime-boost interval regimen, tailored to outbreak response use, with a 4-week regimen. We conducted a phase 1, first-in-human, open-label clinical trial to assess the safety and immunogenicity of MVA-EBO-Z alone and heterologous prime-boost immunization with ChAd3-EBO-Z followed by MVA-EBO-Z at 2 doses in 40 healthy UK volunteers aged 18–50 years. After initial safety assessment in this trial, we conducted a phase 1 trial to assess the 1-week prime-boost interval in 40 Senegalese adults.

## METHODS

### Vaccines

ChAd3-EBO-Z consists of a recombinant replication-deficient adenovirus chimpanzee serotype 3 vector expressing wild-type (WT) Ebola GP from the Zaire strain [6, 7, 10]. MVA-EBO-Z consists of a recombinant, replication-deficient, attenuated vaccinia Ankara virus vector expressing the WT Ebola GP of the Zaire Mayinga strain [18]. The drug substance was manufactured under Good Manufacturing Practice conditions by Emergent BioSolutions in the immortal avian cell line AGE1.CR.Pix.

### Study Participants

The Phase 1a study was conducted in healthy adults between the ages of 18 and 50 years at the Centre for Clinical Vaccinology and Tropical Medicine at the University of Oxford and the Wellcome Trust Clinical Research Facility at Imperial College, London, United Kingdom.

**Figure 1.**
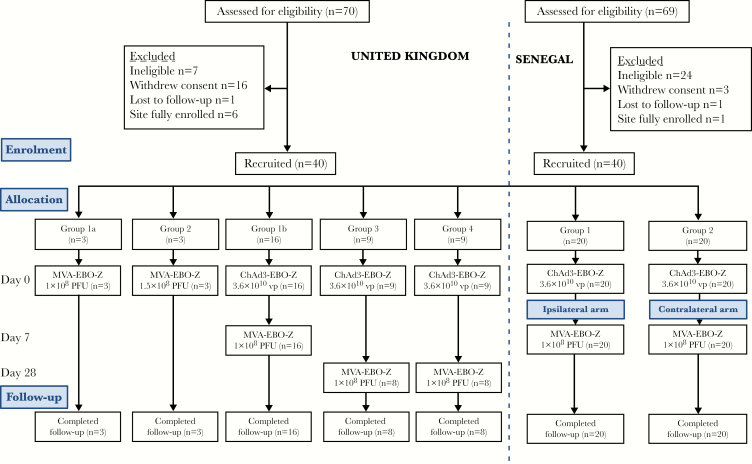
Flowchart of study design and volunteer recruitment: Consolidated Standards of Reporting Trials (CONSORT) diagram of screening, enrollment, vaccination, and follow-up. All vaccinations were given intramuscularly. One volunteer in group 3 and 1 volunteer in group 4 withdrew from the UK study and were replaced; hence, n = 9 were allocated, but only 8 completed follow-up. This was unrelated to vaccination. All volunteers completed the study. There were no withdrawals in the Senegalese study and all volunteers completed the study. Abbreviations: ChAd3-EBO-Z, recombinant chimpanzee adenovirus type 3 vectored Ebola Zaire vaccine; MVA-EBO-Z, modified vaccinia Ankara virus vectored Ebola Zaire vaccine; pfu, plaque-forming units; vp, viral particles.

Subsequently, the phase 1b study was conducted in healthy Senegalese adults aged between 18 and 50 years at the Centre Hospitalier Universitaire le Dantec, Dakar, Senegal. All participants provided written informed consent. Participant flow and study design are summarized in Figure 1. Both studies were conducted according to the principles of the Declaration of Helsinki (2008) and the International Conference on Harmonization Good Clinical Practice guidelines (see the clinical trial protocols in the Supplementary Materials for the full list of inclusion and exclusion criteria).

### Ethics and Regulatory Approval

The study protocol and associated documents for the phase 1a trial were reviewed and approved by the UK National Research Ethics Service (Committee South Central - Oxford A, reference 15/SC/0108), the Medicines and Healthcare Products Regulatory Agency (reference 21584/0341/001-0001), and the Oxford University Clinical Trials and Research Governance team, who independently and externally monitored compliance with Good Clinical Practice guidelines. Vaccine use was authorized by the Genetically Modified Organisms Safety Committee of the Oxford University Hospitals National Health Service Trust (reference number GM462.15.82).

Ethical approval for the phase 1b study was granted in the United Kingdom by the Oxford Tropical Research Ethics Committee (OxTREC reference number 27-15). Ethical and regulatory approvals for this study were also granted in Senegal by the Senegalese Comité National d’Ethique pour la Recherche en Santé and the Senegalese regulatory authority, the Ministry of Health and Social Action Department of Pharmacy and Laboratories.

### Study Design

The phase 1a study was an open-label observational clinical trial assessing the safety and immunogenicity of MVA-EBO-Z alone and the heterologous prime-boost regimen of ChAd3- EBO-Z followed by MVA-EBO-Z. Volunteers were first enrolled into group 1a and group 1b in a staggered manner to receive vaccination with MVA-EBO-Z alone at a dose of 1 × 10^8^ plaque-forming units (PFU) and 1.5 × 10^8^ PFU, respectively. Subsequently, the chief investigator and local safety monitor (LSM) deemed it safe to proceed with vaccinations in groups 2 and 3 after review of the 3 volunteers in group 1a at 72 hours postvaccination. Similarly, the chief investigator and LSM deemed it safe to proceed with vaccinations in group 4 after review of the 3 volunteers in group 1b at 72 hours postvaccination. All vaccinations were administered intramuscularly into the deltoid region of the arm. Most volunteers received both vaccinations in the nondominant arm. Three volunteers (2 in group 2 and 1 in group 3) received their MVA vaccination in the contralateral arm. An independent LSM provided safety oversight. The trial was registered with ClinicalTrials.gov (NCT02451891).

The phase 1b study was a randomized, open-label clinical trial assessing the heterologous prime-boost regimen of ChAd3-EBO-Z followed by MVA-EBO-Z 1 week later, either in the same arm or in the contralateral arm, in healthy Senegalese adults. All vaccinations were administered intramuscularly into the deltoid region of the arm. The trial was registered with ClinicalTrials.gov (NCT02485912). An independent data safety and monitoring board and LSM provided oversight and reviewed preliminary safety data before vaccinations commenced. The LSM also reviewed safety data after the first 5 volunteers had been vaccinated with ChAd3-EBO-Z before the remainder of the volunteers in each group were vaccinated. The trial was monitored by an external organization (Margan Clinical Research Organization). Full details regarding the study conduct are provided in the protocols, which can be found in the Supplementary Materials. Details of the safety analysis are also provided in the Supplementary Materials.

### Assessment of Vaccine Immunogenicity

Antibody responses to vaccination were measured using a standardized enzyme-linked immunosorbent assay against recombinant trimeric Zaire Ebola GP as previously detailed [7], as were neutralizing antibody titers to ChAd3 [23]. Cellular responses were measured using an ex vivo interferon gamma (IFN-γ) enzyme-linked immunospot assay (ELISpot) and intracellular cytokine staining, also as previously described [7]. Further details are given in the Supplementary Materials.

### Statistical Analysis

These were observational and descriptive studies, and the sample size allowed determination of the magnitude of the outcome measures, especially of serious AEs (SAEs) and severe AEs, rather than aiming to obtain statistical significance for differences between groups. Group data show median with interquartile range (IQR) unless otherwise stated. Two groups were compared using Mann–Whitney analyses. Multiple groups were compared using Kruskal–Wallis analyses with Dunn post test for multiple comparisons. For statistical analyses, α = <.05 was considered significant and all *P* values are 2-tailed. All analyses were performed in GraphPad Prism software version 7.

## RESULTS

### Study Population

In the phase 1a study, 70 volunteers were screened for eligibility and 40 were enrolled. Baseline demographics are shown in Supplementary Table 1. One volunteer in group 3 was withdrawn and replaced due to ongoing symptoms of chest pain at the time of the boost vaccination. This volunteer with a previous history of costochondritis was admitted overnight to the hospital with intermittent episodes of sharp nonradiating chest pain associated with exertional dyspnea lasting for 48 hours. This volunteer received vaccination with ChAd3-EBO-Z 3 days before the onset of symptoms. Subsequent cardiac investigations were all normal. The volunteer was withdrawn from the study due to ongoing symptoms of chest pain. A causality of “unlikely” to be related to vaccination was assigned to this SAE. Another volunteer in group 4 withdrew due to logistic reasons and was also replaced. Vaccinations took place between 6 May 2015 and 19 November 2015.

Three volunteers each in group 1a and 1b received MVA-EBO-Z alone at doses of 1 × 10^8^ PFU and 1.5 × 10^8^ PFU, respectively. In groups 2, 3, and 4, 34 volunteers received priming vaccination with ChAd3-EBO-Z at a dose of 3.6 × 10^10^ viral particles. In group 2, 16 volunteers received vaccination with MVA-EBO-Z at a dose of 1 × 10^8^ PFU with a prime-boost interval of 1 week. In each of groups 3 and 4, 8 volunteers were boosted with 1 × 10^8^ PFU and 1.5 × 10^8^ PFU MVA-EBO-Z, respectively 4 weeks after ChAd3-EBO-Z prime.

In the phase 1b study, 69 subjects were screened for eligibility and 40 were enrolled. Baseline demographics are shown in Supplementary Table 2. Vaccinations took place between 2 July 2015 and 14 July 2015. There were no withdrawals and all volunteers completed follow-up. Forty volunteers received ChAd3-EBO-Z followed by MVA-EBO-Z given 1 week later either in the ipsilateral or contralateral arm (20 in each group).

The safety profile of ChAd3-EBO-Z has been described previously [6, 7] and a similar reactogenicity profile was observed after vaccination in the phase 1a study. Most AEs were mild in severity and self-limiting with no severe AEs reported. Four volunteers (11.8%) reported a mild fever postvaccination. Solicited local and systemic AEs related to ChAd3-EBO-Z vaccination in the UK study are shown in Table 1. Solicited local and systemic AEs associated with the MVA-EBO-Z boost vaccination given at a 1-week prime-boost interval are shown in Table 1. The majority of local and systemic AEs were mild in nature. Three volunteers reported fever, which spontaneously resolved within 24 hours. One of these volunteers reported severe fever associated with severe feverishness, fatigue, and malaise, which resolved spontaneously within 24 hours. One volunteer reported severe local erythema on day 4 postvaccination. The majority of solicited AEs in volunteers receiving MVA-EBO-Z at a 4-week interval were mild in nature (Table 1). Increasing the dose of MVA-EBO-Z to 1.5 × 10^8^ PFU did not increase reactogenicity. No fevers or severe AEs were reported in either group.

**Table 1.** Maximum Solicited Local and Systemic Adverse Events Collected for 7 Days After the First Vaccination and 7 Days After the Modified Vaccinia Ankara Virus Vectored Ebola Zaire Vaccine Boost Vaccination in the UK Trial

Symptom and Intensity	MVA-EBO-Z, Group 1a (n = 3)	MVA-EBO-Z, Group 1b (n = 3)	ChAd3-EBO-Z, Groups 2–4 (n = 34)
Solicited adverse events: vaccination 1			
Local			
Pain			
Mild	3 (100)	2 (67)	21 (62)
Moderate	0	1 (33)	4 (12)
Redness			
Mild	2 (67)	0	5 (15)
Swelling			
Mild	0	0	1 (3)
Moderate	0	0	1 (3)
Itching			
Mild	0	0	1 (3)
Moderate	0	0	1 (3)
Warmth			
Mild	1 (33)	0	6 (18)
Moderate	0	0	1 (3)
Systemic			
Fever			
Mild	1 (33)	0	4 (12)
Feverishness			
Mild	0	0	8 (23.5)
Moderate	0	0	3 (9)
Myalgia			
Mild	0	0	2 (6)
Moderate	0	0	3 (9)
Arthralgia			
Mild	1 (33)	0	8 (23.5)
Moderate	0	0	5 (15)
Headache			
Mild	1 (33)	2(67)	9 (26)
Moderate	0	0	6 (18)
Fatigue			
Mild	1 (33)	2 (67)	9 (26)
Moderate	0	0	7 (21)
Nausea			
Mild	0	1 (33)	3 (9)
Moderate	0	0	1 (3)
Malaise			
Mild	0	1 (33)	8 (23.5)
Moderate	0	0	3 (9)
	MVA-EBO-Z, Group 2 (n = 16)	MVA-EBO-Z, Group 3 (n = 8)	MVA-EBO-Z, Group 4 (n = 8)
Solicited adverse events: vaccination 2			
Local			
Pain			
Mild	12 (75)	5 (62.5)	6 (75)
Moderate	2 (12.5)	2 (25)	0
Redness			
Mild	5 (31)	1 (12.5)	0
Moderate	1 (6)	0	0
Severe	1 (6)	0	0
	MVA-EBO-Z, Group 2 (n = 16)	MVA-EBO-Z, Group 3 (n = 8)	MVA-EBO-Z, Group 4 (n = 8)
Swelling			
Mild	3 (19)	0	0
Itching			
Mild	1 (6)	1 (12.5)	0
Warmth			
Mild	4 (25)	3 (37.5)	1 (12.5)
Moderate	1 (6)	0	0
Systemic			
Fever			
Mild	2 (12.5)	0	0
Moderate	0	0	0
Severe	1 (6)	0	0
Feverishness			
Mild	3 (19)	4 (50)	0
Moderate	1 (6)	0	0
Severe	1 (6)	0	0
Myalgia			
Mild	5 (31)	0	0
Moderate	1 (6)	0	0
Arthralgia			
Mild	5 (31)	2 (25)	1 (12.5)
Moderate	2 (12.5)	0	0
Headache			
Mild	7 (44)	3 (37.5)	4 (50)
Moderate	1 (6)	2 (25)	0
Fatigue			
Mild	6 (37.5)	5 (62.5)	2 (25)
Moderate	3 (19)	1 (12.5)	1 (12.5)
Severe	1 (6)	0	0
Nausea			
Mild	4 (25)	3 (37.5)	0
Moderate	1 (6)	0	0
Malaise			
Mild	3 (19)	5 (62.5)	2 (25)
Moderate	3 (19)	0	0
Severe	1 (6)	0	0

Data are presented as No. (%). Frequency is calculated as the number of participants counted once at the time of the worst severity of the event. Intensity categories in which all of the values were zero are not shown. Data are combined for all adverse events for all volunteers receiving the same vaccine at the stated time point.

Abbreviations: ChAd3-EBO-Z, recombinant chimpanzee adenovirus type 3 vectored Ebola Zaire vaccine; MVA-EBO-Z, modified vaccinia Ankara virus vectored Ebola Zaire vaccine.

The reactogenicity profile was significantly milder in the Senegalese compared with the UK cohort, both after the ChAd3-EBO-Z (*P* < .0001) and MVA-EBO-Z vaccinations (*P* < .0001, χ^2^ test). There was no reported fever or severe AEs, and there was a significantly lower proportion of moderate AEs reported. Solicited local and systemic AEs related to vaccination in the Senegalese study are shown in Table 2.

**Table 2.** Maximum Solicited Local and Systemic Adverse Events Collected for 7 Days After Vaccination in the Senegalese Trial

Symptom and Intensity	Group 1 (n = 20)		Group 2 (n = 20)	
	ChAd3-EBO-Z	MVA-EBO-Z	ChAd3-EBO-Z	MVA-EBO-Z
Local				
Pain				
Mild	6 (30)	8 (40)	8 (40)	6 (30)
Moderate	2 (10)	1 (5)	0 (0)	0 (0)
Redness				
Mild	1 (5)	0 (0)	1 (5)	1 (5)
Swelling				
Mild	1 (5)	0 (0)	0 (0)	1 (5)
Itching				
Mild	2 (10)	3 (15)	0 (0)	0 (0)
Warmth				
Mild	2 (10)	4 (20)	1 (5)	0 (0)
Systemic				
Fever	None reported			
Feverishness				
Mild	2 (10)	3 (15)	0 (0)	0 (0)
Myalgia				
Mild	5 (25)	5 (25)	2 (10)	2 (10)
Arthralgia				
Mild	4 (20)	3 (15)	0 (0)	0 (0)
Headache				
Mild	6 (30)	4 (20)	3 (15)	1 (5)
Moderate	1 (5)	0 (0)	0 (0)	0 (0)
Fatigue				
Mild	9 (45)	5 (25)	3 (15)	0 (0)
Nausea				
Mild	1 (5)	1 (5)	1 (5)	0 (0)
Malaise				
Mild	2 (10)	2 (10)	3 (15)	0 (0)
Moderate	1 (5)	0 (0)	0 (0)	0 (0)

Data are presented as No. (%). Frequency is calculated as the number of participants counted once at the time of the worst severity of the event. Intensity categories in which all of the values were zero are not shown. Data are combined for all adverse events for all volunteers receiving the same vaccine at the stated time point.

Abbreviations: ChAd3-EBO-Z, recombinant chimpanzee adenovirus type 3 vectored Ebola Zaire vaccine; MVA-EBO-Z, modified vaccinia Ankara virus vectored Ebola Zaire vaccine.

Unsolicited AEs in the 28 days following vaccination in the UK study were predominantly mild in nature and resolved spontaneously (Supplementary Tables 3 and 4). The majority of laboratory AEs in the UK cohort were grade 1 according to the US Food and Drug Administration toxicity grading scale and resolved spontaneously (Supplementary Table 5). All laboratory AEs in the Senegalese study were mild and resolved spontaneously. There were no SAEs related to vaccination, and no suspected unexpected serious adverse reactions in either study. No individual stopping or group holding rules were activated.

### Humoral Response to Vaccination

**Figure 2.**
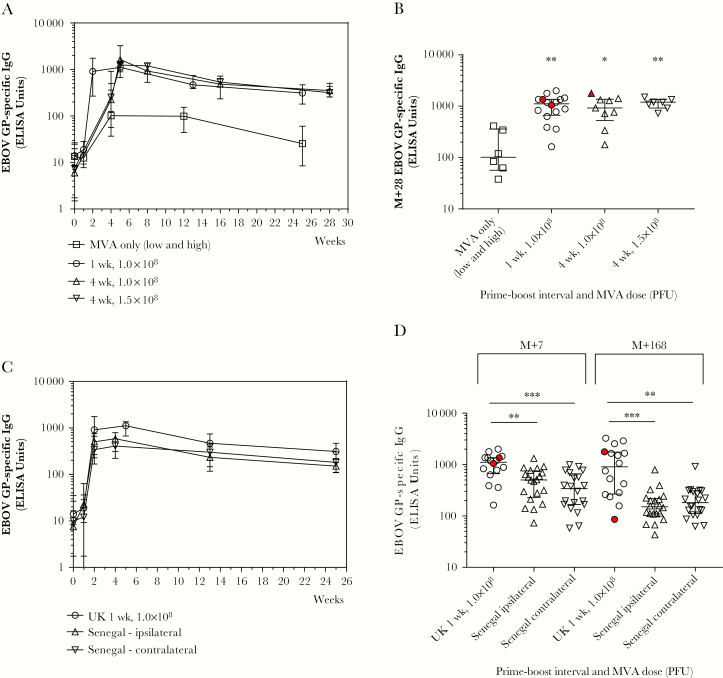
Humoral responses. Ebola virus glycoprotein–specific immunoglobulin G responses. *A*, Median time courses for all groups in the UK cohort. *B*, Comparison of titers at 28 days after modified vaccinia Ankara (MVA) vaccination in all UK groups. Kruskal–Wallis analysis with Dunn posttest comparing prime-boosted groups to the nonboosted group, *P* = .0048. No significant difference across the boosted groups, *P* = .757. *C*, Median time courses for matched groups in the United Kingdom and Senegal (low-dose MVA, 1 week prime-boost interval). *D*, Titers at 1 week and 6 months after MVA vaccination in the United Kingdom and Senegal. Kruskal–Wallis analysis with Dunn posttest comparisons across groups, *P* = .0004 and *P* = .0001, respectively. Bars represent medians and interquartile ranges in all panels. *P < .05, **P < .01, ***P < .001. UK volunteers who received vaccines in a contralateral regimen are highlighted in red. All other UK volunteers received vaccines in an ipsilateral regimen. Abbreviations: EBOV, Ebola virus; ELISA, enzyme-linked immunosorbent assay; GP, glycoprotein; IgG, immunoglobulin G; M+, number of days postvaccination with modified vaccinia Ankara; MVA, modified vaccinia Ankara; PFU, plaque-forming units; UK, United Kingdom.

Individual antibody responses in the UK cohort peaked at either 7 or 28 days post-MVA, referred to hereafter as M+7 or M+28 (Figure 2A). Significant EBOV GP-specific immunoglobulin G (IgG) responses were induced in all groups, including the MVA-only group (*P* = .0313). Responses were compared across groups at M+28 (Figure 2B). Titers in the UK MVA-only group were significantly lower than those in groups that received ChAd3-EBO-Z and MVA-EBO-Z in a prime-boost regimen (*P* = .0048). There were no significant differences between boosted groups at this time point (*P* = .757). Titers induced by ChAd3 and MVA-EBO-Z were comparable to those previously reported for ChAd3 and MVA-BN Filo at the same time point [7]. Antibody responses were well maintained to 168 days post-MVA in boosted groups and there were no significant differences between boosted groups at this time point (*P* = .813). Antibody responses to EBOV GP were compared between matched groups (1 week prime-boost interval, 1.0 × 10^8^ PFU MVA) in the United Kingdom and in Senegal split by ipsilateral or contralateral vaccination (Figure 2C and 2D). Both Senegalese groups had significantly lower EBOV-specific IgG titers at 1 week and 6 months after MVA vaccination compared to the matched group in the UK cohort (*P* = .0004 and *P* = .001, respectively). There were no significant differences between the ipsilateral and contralateral groups within the Senegal cohort at either of these time points, or at any other time point measured.

### Cellular Response to Vaccination

**Figure 3.**
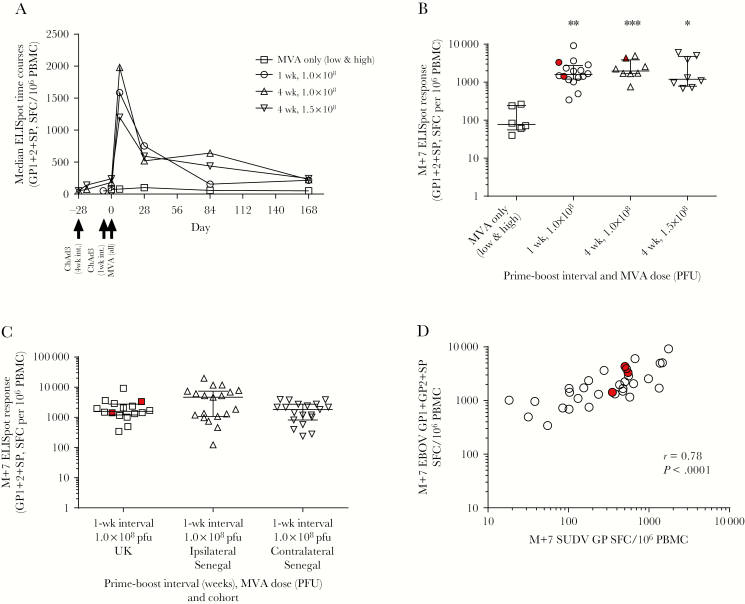
Enzyme-linked immunospot assay (ELISpot) responses to vaccination. *A*, Median time courses of T-cell responses to vaccination in all UK volunteers. *B*, T-cell responses in each UK group at 1 week after modified vaccinia Ankara (MVA) (M+7) (Kruskal–Wallis test, *P* = .0014). *C*, Comparison of peak post-MVA responses (M+7) in Senegalese volunteers vaccinated with recombinant chimpanzee adenovirus type 3 vectored Ebola Zaire vaccine and boosted 1 week later with modified vaccinia Ankara virus vectored Ebola Zaire vaccine in either an ipsilateral or contralateral regimen compared with the dose- and interval-matched regimen in the United Kingdom. Kruskal–Wallis analysis with Dunn posttest, *P* = .237; Mann-Whitney test between ipsilateral and contralateral groups in Senegal, *P* = .0785. Only 3 volunteers across all UK groups received vaccines in a contralateral regimen (highlighted in red); all others received vaccines in an ipsilateral regimen. *D*, Association between the ELISpot responses to Sudan Ebola virus glycoprotein (GP) peptides and summed GP pools for Zaire Ebola virus in prime-boosted UK volunteers at M+7. Spearman *r* = 0.78, *P* < .0001. Black bars on column graphs indicate median and interquartile range. Abbreviations: ChAd3, chimpanzee adenovirus type 3; EBOV, Zaire Ebola virus; ELISpot, enzyme-linked immunospot assay; GP, glycoprotein; M+7, 7 days post– modified vaccinia Ankara; MVA, modified vaccinia Ankara; PFU, plaque-forming units; PBMC, peripheral blood mononuclear cells; SFC, spot-forming cells; SP, signal peptide; SUDV, Sudan Ebola virus; UK, United Kingdom.

The IFN-γ ELISpot responses in the UK vaccinees peaked 7 days after MVA vaccination in all groups and were significantly higher than the MVA-only group with median values of >1000 spot-forming cells (SFCs) per million peripheral blood mononuclear cells (PBMCs) in primed groups compared with 78 in the nonprimed group (Figure 3A and 3B, Kruskal–Wallis test, *P* = .0014). ELISpot responses in prime-boost groups were still significantly higher than the MVA-only group at 3 and 6 months after MVA (Supplementary Figure 1), and there were no significant differences between the boosted groups at any time point. At M+7, there was no significant difference between UK and Senegalese participants (Figure 3C). Administration of MVA by a contralateral or ipsilateral route also did not significantly affect immunogenicity, compared with the dose- and interval-matched regimen in the United Kingdom (Kruskal–Wallis analysis with Dunn posttest, *P* = .237; Mann-Whitney test between ipsilateral and contralateral groups in Senegal, *P* = .0785). Only 3 volunteers across all UK groups received vaccines in a contralateral regimen; all others received vaccines in an ipsilateral regimen. Cross-reactivity with GP peptides from the Sudan Ebola virus (SUDV) was observed at M+7. Homology between EBOV and SUDV GPs is 56% at the amino acid level [24]. Median ELISpot responses to SUDV GP 7 days post-MVA were significantly lower than those to EBOV GP at the same time point (1693 and 406 SFCs per 10^6^ PBMCs, respectively, Mann–Whitney test, *P* < .0001). However, responses to the heterologous GPs were strongly correlated (Figure 3D; *r* = 0.78, *P* < .0001).

**Figure 4.**
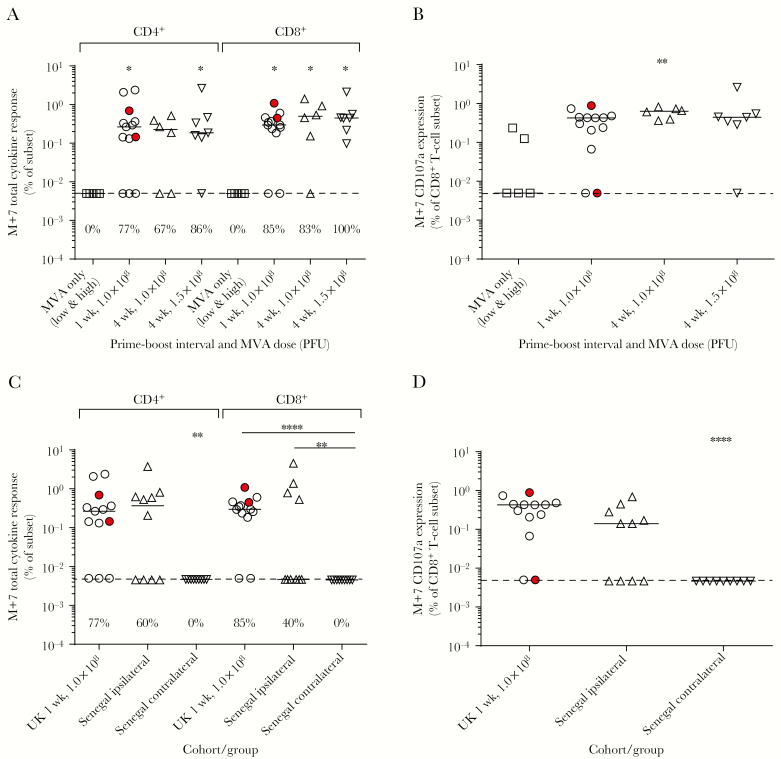
Total cytokine responses. *A*, Total cytokine response measured by flow cytometry with intracellular cytokine staining 7 days postboost according to interval and modified vaccinia Ankara (MVA) dose in the UK cohort (Kruskal–Wallis analysis with Dunn posttest comparisons to the MVA-only group, *P* = .0529 and *P* = .0136 in the CD4^+^ and CD8^+^ subsets, respectively). Percentages above the *x*-axis indicate response rates in each. *B*, Expression of the degranulation marker CD107a in the CD8^+^ subset 7 days postboost in the UK group; Kruskal–Wallis test, *P* = .0152. *C*, Total Ebola glycoprotein–specific cytokine responses in Senegalese groups compared with the matched UK group. Kruskal–Wallis test with Dunn multiple comparisons, *P* = .0085 and *P* < .0001 for the CD4^+^ and CD8^+^ subsets, respectively. *D*, Frequency of CD107a^+^CD8^+^ T cells in Senegalese groups compared to the matched UK group; Kruskal–Wallis test, *P* = .0002. Horizontal bars indicate group medians and dashed lines show the lower limit of detection. UK volunteers who received vaccines in a contralateral regimen are highlighted in red. Intracellular cytokine staining (ICS) data are available for 5 of 6 MVA-only volunteers, 13 of 16 in the UK 1-week 1.0 × 10^8^ group, 6 of 8 in the UK 4-week 1.0 × 10^8^ group, 7 of 8 in the UK 4-week 1.5 × 10^8^ group, 10 of 20 in the Senegal ipsilateral group, and 9 of 20 in the Senegal contralateral group. Data are not present if there were too few fresh cells remaining after enzyme-linked immunospot assay to conduct ICS, if too few events were obtained, or the sample failed assay quality control. Asterisks indicate level of significance between groups calculated using Dunn posttest comparison after Kruskal–Wallis analysis. *P < .05, **P < .01, ****P < .0001. Abbreviations: M+7, 7 days post– modified vaccinia Ankara; MVA, modified vaccinia Ankara; PFU, plaque-forming units; UK, United Kingdom.

Cytokine responses were determined by intracellular staining and flow cytometry 7 days after MVA vaccination. The total antigen-specific cytokine response (frequency of CD4^+^ or CD8^+^ T cells secreting IFN-γ, interleukin 2, or tumor necrosis factor–α in Ebola GP–stimulated PBMCs minus that in unstimulated cells) was compared across all UK groups (Figure 4A). Individuals who received MVA alone showed no detectable cytokine responses. In the CD4^+^ T-cell compartment, all boosted groups had a response rate of at least 67% and a median response of at least 0.19%. In the CD8^+^ T-cell compartment, the response rate was >80% in all boosted groups, with median responses >0.30%. Expression of the degranulation marker CD107a by CD8^+^ T cells was observed in almost all boosted individuals and in 2 individuals given only MVA (Figure 4B).

Total cytokine responses to Ebola GP were compared between the Senegalese cohort and the matched UK group (Figure 4C). CD4^+^ responses were not above background in the contralateral group of the Senegal cohort (0.005%), while the ipsilateral group had median responses (0.37% [IQR, 0.005%–0.67%]) that were not significantly different to the United Kingdom (0.27% [IQR, 0.07%–0.83%]). CD8^+^ frequencies were similarly undetectable in the contralateral group of the Senegal cohort (0.005%), while the ipsilateral group had median responses (0.005% [IQR, 0.005%–0.93%]) that were not significantly different to the United Kingdom (0.29% [IQR, 0.2%–0.46%]). For both the CD4^+^ and CD8^+^ subsets, cytokine responses in the Senegal contralateral group were significantly lower than both other groups (CD4^+^, *P* = .009; CD8^+^, *P* < .0001, Kruskal–Wallis test). The frequency of CD107a^+^CD8^+^ T cells was also comparable in the United Kingdom and ipsilateral group of the Senegal cohort, whereas no CD107a expression was detected in the contralateral group (Figure 4D). Proportions of cells producing different combinations of cytokines were additionally compared across boosted groups (Supplementary Figure 2).

### Antivector Immunity

Preexisting neutralizing antibodies to the ChAd3 vector were measured in the Senegal cohort at baseline. Responses were detectable in all participants but were low in magnitude (geometric mean titer, 121.4 [95% confidence interval, 90.6–162.8]) and only 9 of 40 participants had a titer >200. Anti-ChAd3 neutralizing antibody titer at baseline did not correlate with any measure of postvaccination immunogenicity (Table 3).

**Table 3.** Association Between Preexisting Antivector Immunity and Measures of Vaccine Immunogenicity Measured by Enzyme-Linked Immunosorbent Assay and Enzyme-Linked Immunospot Assay at Different Time Points

Measure	ChAd3 nAb Titer vs M+7 ELISpot	ChAd3 nAb Titer vs M+7 ELISA	ChAd3 nAb Titer vs M+28 ELISA	ChAd3 nAb Titer vs M+90 ELISA	ChAd3 nAb Titer vs M+180 ELISA
Number of XY pairs	38	40	40	39	40
Spearman *r*	0.17	–0.14	–0.19	–0.27	–0.21
95% CI	–.17 to .47	–.44 to .19	–.48 to .13	–.55 to .06	–.49 to .12
*P* value (2-tailed)	0.31	0.38	0.23	0.1	0.20
Significant? (α = .05)	No	No	No	No	No

Abbreviations: ChAd3, recombinant chimpanzee adenovirus type 3; CI, confidence interval; ELISA, enzyme-linked immunosorbent assay; ELISpot, enzyme-linked immunospot assay; M+, number of days postvaccination with modified vaccinia Ankara; nAb, neutralizing antibody.

## DISCUSSION

Monovalent MVA-EBO-Z, the first to be manufactured in an immortalized cell line and administered to humans, was safe and well tolerated in phase 1 trials in UK and West African adults. Adverse events after ChAd3-EBO-Z were predominantly mild in nature, in keeping with previous studies [6, 7]. MVA-EBO-Z administered at a dose of 1.5 × 10^8^ PFU did not increase the reactogenicity compared to 1 × 10^8^ PFU, and this vaccine showed significantly reduced reactogenicity in African adults.

MVA-EBO-Z given as a boost following a ChAd3-EBO-Z vaccine elicited both humoral and cell-mediated immune responses, comparable to a multivalent MVA-BN Filo expressing both Zaire and Sudan Ebola virus GPs [7]. T-cell responses peak at M+7 and antibody responses at M+28; therefore, a very short prime-boost interval of 1 week appears suitable for use in a ring vaccination strategy or for outbreak control. There were no significant differences in Ebola GP–specific IgG titers or median ELISpot responses between groups 2, 3, and 4 at any of the time points. The significant induction of cellular immunity (and particularly IFN-γ–producing CD8^+^ T cells) may be beneficial for long-term protection against EVD [18]. In this respect, a heterologous prime-boost regimen with the viral vectored vaccines ChAd3-EBO-Z and MVA-EBO-Z may be preferable for the durable protection of healthcare workers and populations susceptible to sporadic outbreaks to rVSV-ZEBOV, which does not induce significant Ebola-specific IFN-γ responses [25].

Humoral, but not cellular, immunogenicity postimmunization was significantly reduced in Senegalese volunteers compared to the UK cohort. Reduced antibody responses to vaccination in developing countries have been observed previously, although most often in children and infants, and this phenomenon is likely multifactorial with contributing factors including an increased burden of pathogen exposure, genetic differences, microflora composition, and nutritional status [26–28]. Despite this, antibody responses in the Senegalese cohort were durable, remaining significantly above baseline 6 months after vaccination and although some preexisting immunity to the ChAd3 vector was detected, this did not correlate with reduced antibody responses.

There were no significant differences in antibody or T-cell responses measured by ELISpot between the ipsilateral and contralateral regimens. Significant differences were apparent in the cytokine responses measured by intracellular cytokine staining, with the ipsilateral regimen inducing significantly lower frequencies of antigen-specific cytokine-secreting T cells and a distinct cytokine profile. This is an intriguing observation, perhaps dependent on which lymph nodes the boosting vector drains to, and which clearly warrants further investigation in future vaccine trials in both UK and African populations.

In conclusion, our study shows that the heterologous prime-boost immunization with ChAd3-EBO-Z followed by MVA-EBO-Z was safe and immunogenic and supports further testing in populations at risk of EVD in phase 2 and phase 3 studies. This was also the first trial of any MVA biomanufactured on an immortalized cell line with capacity for very large-scale manufacturing, high production yields, and lower cost of goods compared to other MVA production technologies. The AE profile was very similar to that of MVA-vectored vaccines manufactured in primary chicken embryo cells, supporting the use of this vaccine production method for other MVA-vectored vaccines.

## Supplementary Data

Supplementary materials are available at *The Journal of Infectious Diseases* online. Consisting of data provided by the authors to benefit the reader, the posted materials are not copyedited and are the sole responsibility of the authors, so questions or comments should be addressed to the corresponding author.

Supplementary Figure 1

Supplementary Figure 2

Supplementary Table 1

Supplementary Table 2

Supplementary Table 3

Supplementary Table 4

Supplementary Table 5

Supplementary Text

## Notes

***Acknowledgments.*** We thank all the study participants in the United Kingdom and in Senegal; the members of the data safety monitoring board; Dr Irma Eneida Almeida Dos Santos and Dr Daouda Coly for clinical support; Dr Mouhamadou Ndiaye, Astou Gueye, and Aminata Dia, the study pharmacists in Senegal; and Papa Alassane Diaw, Tidiane Siby, Khadidiatou Mody Ndiaye, Khadidiatou Diagne, Boly Ameth Niang, Yaye Mareme Niang, Serigne Mbacké Diop, Mme Ndéye Astou Fall, Daouda Gueye, Mody Ndiaye, and Ibrahima Gueye for support with the Senegal study. We also acknowledge the contribution of Eric Balsley, Richard Welch, Vijay Yabannavar, and Ian Henderson at Emergent Biosolutions for manufacture of the MVA-EBO-Z vaccine. The EBO-Z GP trimeric protein was produced and donated by Dr Jing Jin and Professor Simon Draper at the Jenner Institute. The ChAd3 vaccine was provided by the Vaccine Research Center of the National Institute of Allergy and Infectious Diseases and GlaxoSmithKline.

***Disclaimer.*** This paper presents independent research supported by the National Institute for Health Research (NIHR) Clinical Research Facility at Imperial College Healthcare National Health Service (NHS) Trust. The views expressed are those of the authors and not necessarily those of the NHS, the NIHR, or the Department of Health.

***Financial support.*** This work was supported by the Wellcome Trust, the UK Medical Research Council, the UK Department for International Development, and the UK NIHR Oxford Biomedical Research Centre. The Good Manufacturing Practice biomanufacture of MVA-EBO-Z and the UK clinical trial was supported by an Enhancement Award to a Wellcome Trust Strategic Award (to A. V. S. H. as principal investigator) co-funded by the Medical Research Council, the Department for International Development, and the European and Developing Countries Clinical Trials Partnership (grant number 106325/Z/14/A), with additional support from the NIHR Oxford Biomedical Research Centre. The Senegal trial was largely funded by a European Commission Horizon 2020 program award EbolaVac (www.ebolavac.eu grant agreement 666085), which provided additional resources for the MVA-EBO-Z biomanufacture. This study was also supported by GlaxoSmithKline Biologicals SA.

***Potential conflicts of interest.*** F. R. and W. R. B. are employees of GSK and own restricted shares of the company. S. C. G., K. E., and A. V. S. H. are named inventors on patents relating to viral vector vaccines for malaria and other diseases. F. R. and W. R. B. are employees of GSK, which is developing vectored vaccines for Ebola and other diseases. All other authors report no potential conflicts of interest. All authors have submitted the ICMJE Form for Disclosure of Potential Conflicts of Interest. Conflicts that the editors consider relevant to the content of the manuscript have been disclosed.

Presented in part: 65th Annual Meeting of the American Society of Tropical Medicine and Hygiene, Atlanta, Georgia, November 2016; and European Congress of Clinical Microbiology and Infectious Diseases, Amsterdam, The Netherlands, April 2016.
