# Supplementary material for: Safety and Immunogenicity of a Heterologous Prime-Boost Ebola Virus Vaccine Regimen in Healthy Adults in the United Kingdom and Senegal
Source: J Infect Dis. 2018 Nov 8;219(8):1187–97. doi: 10.1093/infdis/jiy639 (PMC6452431; doi:10.1093/infdis/jiy639)
Supplement: Supplementary Figure 1 [file jiy639_suppl_supplementary_figure1.docx]

**Supplementary Material**

**Durability of T cell responses**

Supplementary Figure 1. T cell responses to Ebola GP measured by ELISpot A) three months after MVA, M+84 (Kruskal-Wallis P=0.0002) and B) six months after MVA, M+168 (Kruskal-Wallis P=0.0114). Asterisks indicate significant differences compared to the non-primed (MVA only) group. Kruskal-Wallis analyses with Dunn’s multiple comparisons * p<0.05, ** p<0.01, *** P<0.001.
