# Supplementary material for: Safety and Immunogenicity of a Heterologous Prime-Boost Ebola Virus Vaccine Regimen in Healthy Adults in the United Kingdom and Senegal
Source: J Infect Dis. 2018 Nov 8;219(8):1187–97. doi: 10.1093/infdis/jiy639 (PMC6452431; doi:10.1093/infdis/jiy639)
Supplement: Supplementary Figure 2 [file jiy639_suppl_supplementary_figure2.docx]

**Supplementary Figure 2**

**
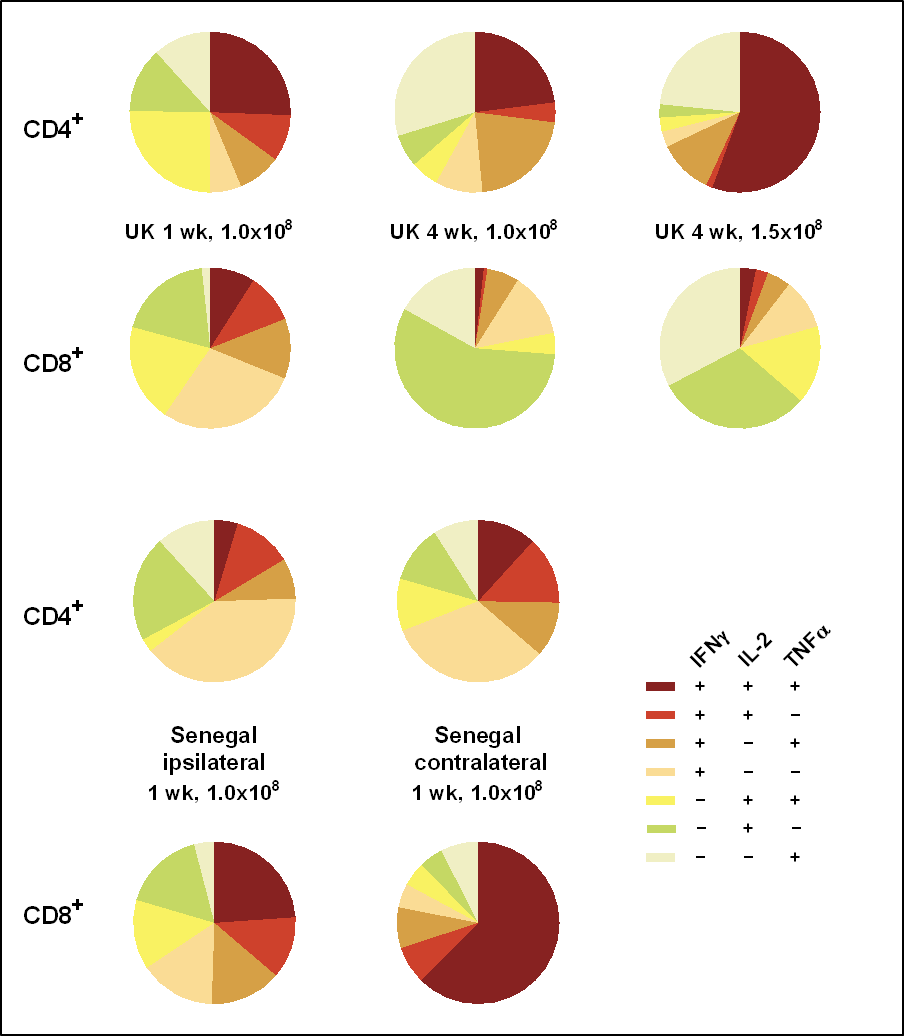
**

Supplementary Figure 2. Cytokine profiles of antigen-specific CD4^+^ and CD8^+^ T cells in the UK cohort grouped by prime-boost interval and MVA dose in pfu and in the Senegalese cohort grouped by regimen. Frequencies of CD4^+^ and CD8^+^ T cell subsets expressing different combinations of IFNγ, IL-2 and TNFα in the Ebola GP-stimulated sample (after subtraction of background) were expressed as a proportion of the total antigen-specific cytokine response and group geomeans are displayed.
