# Supplementary material for: Safety and Immunogenicity of a Heterologous Prime-Boost Ebola Virus Vaccine Regimen in Healthy Adults in the United Kingdom and Senegal
Source: J Infect Dis. 2018 Nov 8;219(8):1187–97. doi: 10.1093/infdis/jiy639 (PMC6452431; doi:10.1093/infdis/jiy639)
Supplement: Supplementary Table 1 [file jiy639_suppl_supplementary_table1.docx]

|  | | | | |
| --- | --- | --- | --- | --- |
|  | **Group 1 (n=6)** | **Group 2 (n=16)** | **Group 3 (n=9)** | **Group 4 (n=9)** |
|  | ***Number (percent)*** | | | |
| Sex  Male  Female | 3 (50)  3 (50) | 6 (37.5)  10 (62.5) | 3 (33)  6 (67) | 5 (56)  4 (44) |
| Age  18-20 yrs  21-30 yrs  31-40 yrs  41-50 yrs  Mean (yrs) | 0  5 (83)  0  1 (17)  30 | 0  9 (56)  3 (19)  4 (25)  33 | 0  6 (67)  2 (22)  1 (11)  30 | 0  6 (67)  2 (22)  1 (11)  30 |
| Race*  White  Black  Asian  Mixed  Other | 6 (100)  0  0  0  0 | 13 (81)  1 (6)  0  2 (13)  0 | 8 (89)  0  0  1 (11)  0 | 7 (78)  0  0  1 (11)  1 (11) |
| Body-mass index^%^  < 18.5  18.5-24.9  25-29.9  ≥ 30  Mean | 1 (17)  2 (33)  2 (33)  1 (17)  25 | 0  6 (38)  5 (31)  5 (31)  27 | 1 (11)  6 (67)  2 (22)  0  22 | 0  5 (56)  3 (33)  1 (11)  24 |

**Supplementary Table 1. Demographics and baseline characteristics of volunteers enrolled in the UK trial.** *There were no significant differences between the study groups. *Race was self-reported. ^%^The body-mass index is the weight in kilograms divided by the square of the height in meters.*
