# Supplementary material for: Safety and Immunogenicity of a Heterologous Prime-Boost Ebola Virus Vaccine Regimen in Healthy Adults in the United Kingdom and Senegal
Source: J Infect Dis. 2018 Nov 8;219(8):1187–97. doi: 10.1093/infdis/jiy639 (PMC6452431; doi:10.1093/infdis/jiy639)
Supplement: Supplementary Table 2 [file jiy639_suppl_supplementary_table2.docx]

|  | | |
| --- | --- | --- |
|  | **Group 1 (n=20)** | **Group 2 (n=20)** |
|  | ***Number (percent)*** | |
| Sex  Male  Female | 13 (65)  7 (35) | 16 (80)  4 (20) |
| Age  18-20 yrs  21-30 yrs  31-40 yrs  41-50 yrs  Mean (yrs) | 7 (35)  9 (45)  4 (20)  0  26.2 | 2 (10)  8 (40)  8 (40)  2 (10)  30.5 |
| Race  White  Black  Asian  Mixed  Other | 0  20 (100)  0  0  0 | 0  20 (100)  0  0  0 |
| Body-mass index^%^  < 18.5  18.5-24.9  25-29.9  ≥ 30  Mean | 1 (5)  13 (65)  3 (15)  2 (10)  23.4 | 0  14 (70)  4 (20)  2 (10)  24 |

**Supplementary Table 2. Demographics and baseline characteristics of volunteers enrolled in the Senegalese trial.** *There were no significant differences between the study groups. ^%^The body-mass index is the weight in kilograms divided by the square of the height in meters. Height was not recorded in one Group 1 volunteer.*
