# Supplementary material for: Safety and Immunogenicity of a Heterologous Prime-Boost Ebola Virus Vaccine Regimen in Healthy Adults in the United Kingdom and Senegal
Source: J Infect Dis. 2018 Nov 8;219(8):1187–97. doi: 10.1093/infdis/jiy639 (PMC6452431; doi:10.1093/infdis/jiy639)
Supplement: Supplementary Table 3 [file jiy639_suppl_supplementary_table3.docx]

|  | | | | | | | |
| --- | --- | --- | --- | --- | --- | --- | --- |
|  |  | **Vaccination 1** | | | **Vaccination 2** | | |
| **Symptom** | **Intensity** | **MVA EBO Z Group 1a**  **(n=3)** | **MVA EBO Z Group 1b (n=3)** | **ChAd3 EBO Z Groups 2-4 (n=34)** | **MVA EBO Z Group 2**  **(n=16)** | **MVA EBO Z Group 3**  **(n=8)** | **MVA EBO Z Group 4**  **(n=8)** |
|  |  | ***Number (percent)*** | | | | | |
| Abdominal pain upper | Mild  Severe | 0  0 | 0  1 (33) | 0  0 | 1 (6)  0 | 0  0 | 0  0 |
| Anxiety | Mild | 0 | 0 | 1 (3) | 0 | 0 | 0 |
| Arthralgia | Mild | 0 | 0 | 1 (3) | 0 | 0 | 0 |
| Back pain | Mild | 0 | 0 | 0 | 1(6) | 0 | 0 |
| Bruxism | Mild | 0 | 0 | 1 (3) | 0 | 0 | 0 |
| Chest pain | Severe | 0 | 0 | 1 (3) | 0 | 0 | 0 |
| Contusion | Mild | 0 | 0 | 1 (3) | 0 | 0 | 0 |
| Cough | Mild | 0 | 0 | 0 | 1 (6) | 0 | 0 |
| Diarrhoea | Mild | 0 | 0 | 1 (3) | 0 | 0 | 0 |
| Discomfort | Mild | 0 | 0 | 0 | 0 | 1 (12.5) | 0 |
| Dizziness | Mild  Moderate  Severe | 1 (33)  0  0 | 0  0  0 | 0  0  0 | 0  1 (6)  1 (6) | 0  0  0 | 0  0  0 |
| Dyspnoea | Moderate | 0 | 0 | 1 (3) | 0 | 0 | 0 |
| Eczema | Mild | 0 | 0 | 1 (3) | 0 | 0 | 0 |
| Epistaxis | Mild | 0 | 0 | 0 | 0 | 1 (12.5) | 0 |
| Erythema | Mild | 0 | 0 | 0 | 0 | 1 (12.5) | 0 |
| Fatigue | Mild  Moderate | 0  0 | 0  0 | 1 (3)  0 | 0  1 (6) | 0  0 | 1 (12.5)  0 |
| Hangover | Moderate | 0 | 0 | 1 (3) | 0 | 0 | 0 |
| Headache | Mild  Moderate | 0  0 | 0  0 | 2 (6)  0 | 0  0 | 1 (12.5)  1 (12.5) | 1 (12.5)  1 (12.5) |
| Influenza like illness | Mild  Moderate | 1 (33)  0 | 0  0 | 0  0 | 0  1 (6) | 1 (12.5)  0 | 0  0 |
| Injection site swelling | Mild | 0 | 0 | 1 (3) | 1 (6) | 0 | 0 |
| Insomnia | Mild | 0 | 0 | 1 (3) | 0 | 0 | 0 |
| Lymphadenopathy | Mild  Moderate | 0 | 0  0 | 2 (6)  1 (3) | 0  0 | 1 (12.5)  0 | 0  0 |
| Malaise | Moderate | 0 | 0 | 0 | 1 (6) | 0 | 0 |
| Migraine | Mild  Moderate  Severe | 0  0  0 | 0  1 (33)  0 | 1 (3)  0  0 | 0  1 (6)  0 | 0  0  1 (12.5) | 0  0  0 |
| Mood swings | Moderate | 0 | 0 | 0 | 1 (6) | 0 | 0 |
| Musculoskeletal stiffness | Mild | 1 (33) | 0 | 0 | 0 | 0 | 0 |
| Myalgia | Mild | 0 | 0 | 2 (6) | 0 | 0 | 1 (12.5) |
| Nausea | Moderate | 0 | 0 | 1 (3) | 0 | 0 | 1 (12.5) |
| Nasopharyngitis | Mild  Moderate | 0  0 | 1 (33)  0 | 0  0 | 0  0 | 0  1 (12.5) | 0  0 |
| Neck pain | Mild  Moderate | 0  0 | 0  0 | 1 (3)  0 | 0  1 (6) | 0  0 | 0  1 (12.5) |
| Oropharyngeal pain | Mild | 1 (33) | 0 | 1 (3) | 1 (6) | 0 | 0 |
| Pain | Mild | 0 | 0 | 2 (6) | 0 | 0 | 0 |
| Pain in extremity | Mild | 0 | 0 | 0 | 1 (6) | 0 | 0 |
| Rash | Mild | 0 | 0 | 0 | 0 | 0 | 1 (12.5) |
| Rash papular | Mild | 0 | 0 | 1 (3) | 0 | 0 | 0 |
| Seasonal allergy | Mild  Moderate | 0  1 (33) | 0  0 | 1 (3)  1 (3) | 1 (6)  0 | 0  0 | 0  1 (12.5) |
| Sinus congestion | Mild | 0 | 0 | 0 | 1 (6) | 0 | 0 |
| Stomatitis | Mild | 0 | 0 | 0 | 0 | 0 | 1 (12.5) |
| Swelling face | Moderate | 1 (33) | 0 | 0 | 0 | 0 | 0 |
| Syncope | Mild | 0 | 0 | 0 | 0 | 1 (12.5) | 0 |
| Thirst | Mild | 0 | 0 | 1 (3) | 0 | 0 | 0 |
| Vomiting | Moderate | 0 | 0 | 1 (3) | 0 | 0 | 0 |

**Supplementary Table 3. The frequency of all unsolicited AEs reported in the 28 days following vaccination in the UK trial.** *Frequency is calculated as the number of subjects counted once at worst severity. Rows with all zero values are not shown. AEs have been classified according to the Medical Dictionary for Regulatory Activities (MedDRA) at the Preferred Term level*
