# Supplementary material for: Safety and Immunogenicity of a Heterologous Prime-Boost Ebola Virus Vaccine Regimen in Healthy Adults in the United Kingdom and Senegal
Source: J Infect Dis. 2018 Nov 8;219(8):1187–97. doi: 10.1093/infdis/jiy639 (PMC6452431; doi:10.1093/infdis/jiy639)
Supplement: Supplementary Table 4 [file jiy639_suppl_supplementary_table4.docx]

|  | | | | | | | |
| --- | --- | --- | --- | --- | --- | --- | --- |
|  |  | **Vaccination 1** | | | **Vaccination 2** | | |
| **Symptom** | **Intensity** | **MVA EBO Z Group 1a (n=3)** | **MVA EBO Z Group 1b (n=3)** | **ChAd3 EBO Z Groups 2-4 (n=34)** | **MVA EBO Z Group 2**  **(n=16)** | **MVA EBO Z Group 3**  **(n=8)** | **MVA EBO Z Group 4**  **(n=8)** |
|  |  | ***Number (percent)*** | | | | | |
| Arthralgia | Mild | 0 | 0 | 1 (3) | 0 | 0 | 0 |
| Back pain | Mild | 0 | 0 | 0 | 1 (6) | 0 | 0 |
| Cough | Mild | 0 | 0 | 0 | 1 (6) | 0 | 0 |
| Diarrhoea | Mild | 0 | 0 | 1 (3) | 0 | 0 | 0 |
| Discomfort | Mild | 0 | 0 | 0 | 0 | 1 (12.5) | 0 |
| Dizziness | Mild  Severe | 1 (33)  0 | 0  0 | 0  0 | 0  1 (6) | 0  0 | 0  0 |
| Erythema | Mild | 0 | 0 | 0 | 0 | 1 (12.5) | 0 |
| Fatigue | Mild  Moderate | 0  0 | 0  0 | 1 (3)  0 | 0  1 (6) | 0  0 | 0  0 |
| Headache | Mild | 0 | 0 | 1 (3) | 0 | 1 (12.5) | 0 |
| Injection site swelling | Mild | 0 | 0 | 1 (3) | 1 (6) | 0 | 0 |
| Insomnia | Mild | 0 | 0 | 1 (3) | 0 | 0 | 0 |
| Lymphadenopathy | Mild | 0 | 0 | 2 (6) | 0 | 1 (12.5) | 0 |
| Malaise | Moderate | 0 | 0 | 0 | 1 (6) | 0 | 0 |
| Musculoskeletal stiffness |  | 1 (33) | 0  0 | 0 | 0 | 0 | 0 |
| Myalgia | Mild | 0 | 0 | 2 (6) | 0 | 0 | 0 |
| Nasopharyngitis | Mild | 0 | 1 (33) |  |  |  |  |
| Neck pain | Mild  Moderate | 0  0 | 0  0 | 1 (3)  0 | 0  1 (6) | 0  0 | 0  0 |
| Oropharyngeal pain | Mild | 1 (33) | 0 | 1 (3) | 1 (6) | 0 | 0 |
| Pain | Mild | 0 | 0 | 2 (6) | 0 | 0 | 0 |
| Pain in extremity | Mild | 0 | 0 | 0 | 1 (6) | 0 | 0 |
| Rash popular | Mild | 0 | 0 | 1 (3) | 0 | 0 | 0 |
| Sinus congestion | Mild | 0 | 0 | 0 | 1 (6) | 0 | 0 |
| Thirst | Mild | 0 | 0 | 1 (3) | 0 | 0 | 0 |

**Supplementary Table 4. Unsolicited Adverse Events related to vaccination. The frequency of all unsolicited AEs deemed definitely, probably and possibly related to vaccination reported in the 28 days following vaccination in the UK trial.** *Frequency is calculated as the number of subjects counted once at worst severity. Rows with all zero values are not shown. AEs have been classified according to the Medical Dictionary for Regulatory Activities (MedDRA) at the Preferred Term level.*
