# Supplementary material for: Safety and Immunogenicity of a Heterologous Prime-Boost Ebola Virus Vaccine Regimen in Healthy Adults in the United Kingdom and Senegal
Source: J Infect Dis. 2018 Nov 8;219(8):1187–97. doi: 10.1093/infdis/jiy639 (PMC6452431; doi:10.1093/infdis/jiy639)
Supplement: Supplementary Table 5 [file jiy639_suppl_supplementary_table5.docx]

|  | | | | | | |
| --- | --- | --- | --- | --- | --- | --- |
|  | **Vaccination** **1** | | | **Vaccination 2** | | |
| **Lab AE and intensity** | **MVA EBO Z Group 1a (n=3)** | **MVA EBO Z Group 1b**  **(n=3)** | **ChAd3 EBO Z Groups 2-4 (n=34)** | **MVA EBO Z Group 2**  **(n=16)** | **MVA EBO Z Group 3**  **(n=8)** | **MVA EBO Z Group 4**  **(n=8)** |
|  | ***Number (percent)*** | | | | | |
| Anaemia  Grade 1  Grade 2 | 0  0 | 0  0 | 3 (9)  0 | 1 (6)  0 | 0  1 (12.5) | 1 (12.5)  0 |
| Elevated WBC  Grade 1 | 0 | 0 | 1 (3) | 1 (6) | 0 | 0 |
| Low WBC  Grade 1 | 0 | 0 | 2 (6) | 0 | 0 | 0 |
| Neutropaenia  Grade 1  Grade 2 | 0  0 | 0  0 | 2 (6)  1 (3) | 2 (12.5) | 1 (12.5) | 1 (12.5) |
| Lymphopaenia  Grade 1  Grade 2 | 0  0 | 0  0 | 2 (6)  0 | 0  1 (6) | 1 (12.5)  0 | 0  0 |
| Eosinophillia  Grade 1 | 0 | 1 (33) | 0 | 0 | 0 | 0 |
| Thrombocytopaenia  Grade 1 | 1 (33) | 0 | 0 | 0 | 0 | 0 |
| Hyperbilirubinaemia  Grade 2 | 0 | 0 | 1 (3) | 0 | 0 | 0 |
| Elevated ALT  Grade 1 | 0 | 0 | 0 | 0 | 0 | 1 (12.5) |
| Elevated Urea  Grade 1 | 0 | 0 | 1 (3) | 0 | 0 | 0 |
| Hypokalaemia  Grade 1  Grade 2  Grade 3 | 0  0  0 | 0  0  0 | 3 (9)  0  0 | 3 (19)  1 (6)  1 (6) | 0  0  0 | 0  0  0 |
| Hyponatraemia  Grade 1 | 0 | 0 | 0 | 0 | 0 | 1 (12.5) |
|  |  |  |  |  |  |  |
| **Supplementary Table 5. Laboratory adverse events. The frequency of all laboratory adverse events in the UK trial.** *Adverse events are defined as a deviation from the baseline result collected pre-vaccination on Day 0.* | | | | | | |
