# Supplementary material for: Safety and Immunogenicity of a Heterologous Prime-Boost Ebola Virus Vaccine Regimen in Healthy Adults in the United Kingdom and Senegal
Source: J Infect Dis. 2018 Nov 8;219(8):1187–97. doi: 10.1093/infdis/jiy639 (PMC6452431; doi:10.1093/infdis/jiy639)
Supplement: Supplementary Text [file jiy639_suppl_supplementary_text.docx]

**Supplementary Text**

**Cytokine profiles**

Frequencies of CD4^+^ and CD8^+^ T cell subsets expressing combinations of IFNγ, IL-2 and TNFα in the Ebola GP-stimulated sample (after background subtraction) were expressed as a proportion of the total antigen-specific cytokine response and displayed as pie charts to compare the cytokine profiles of each of the boosted groups (Figure 6, group geomeans). Within CD4^+^ T cells triple positive and IL-2^+^TNFα^+^ double positives are codominant in the group boosted after one week, while the proportion of TNFa^+^ single positives increases with an increased prime-boost interval and triple positives are dominant in the high dose MVA group. Within CD8^+^ T cells single positive IFNγ predominates in the one week interval group. In the four week interval groups single positive IL-2^+^ or TNFa^+^ dominate. Both Senegalese groups had lower proportions of triple positive and higher proportions of single positive IFNg^+^ CD4^+^ T cells than the matched UK group. The cytokine profiles of the CD8^+^ T cells were comparable between the UK and the ipsilateral Senegalese group but the contralateral group had a profile dominated by triple positive cells.

**Supplementary Methods**

**Safety Analysis**

Participants were observed for 60 minutes after vaccination. All participants in the Phase Ia trial were given access to an electronic diary card on which to record all solicited symptoms for seven days after vaccination and unsolicited symptoms for 28 days after vaccination. Follow-up visits were scheduled for days 1, 3, 7 and 28 after each vaccination; in addition, visits were conducted at 84 and 168 days after the final vaccination. A review of symptoms occurred at each follow-up visit; in addition, testing that included a full blood count, urea and electrolytes, liver enzymes, activated partial thromboplastin time, prothrombin time, and fibrinogen was undertaken at days 3, 7 and 28 after vaccinations. In the Phase Ib study, volunteers were visited at home daily for six days after each vaccination by a field worker for assessment and recording of any solicited and unsolicited AEs in diary cards. They were also seen in clinic at days 14, 28, 58, 90 and 180 for a review of symptoms and safety bloods including full blood count, urea and electrolytes and liver enzymes were performed. Severity grading of adverse events was conducted according to predefined criteria, which are stated in the protocol.

**Standardised Glycoprotein IgG ELISA**

Serum samples were taken for analysis of humoral immunogenicity at baseline (day 0, D0), day of MVA vaccination (M+0) and at 7, 28, 84 and 168 days post-MVA (M+7, M+28, M+84 and M+168 in the UK and at D0, D7, D14 (M+7), D28 (M+21), D90 (M+83) and D180 (M+173) in Senegal. Total IgG responses were measured by standardised EBOV GP ELISA as previously described [1].

**Neutralising antibody assay for ChAd3**

ChAd3 neutralising antibody (NAb) titres were assayed as previously described using a secreted alkaline phosphatase (SEAP) assay [2]. Briefly, 8 x 10^4^ HEK293 cells per well were seeded in a 96-well-plate for 1 day. A fixed and pre-determined amount of SEAP-expressing ChAd3 was pre-incubated for 1 hour at 37 °C alone or with serial dilutions of heat-inactivated serum from trial volunteers, then was added to the 80-90% confluent HEK293 cells for 1 hour at 37 °C, after which the supernatant was replaced with 10% FBS in DMEM. SEAP activity was measured in the supernatant after 24 ± 2 hours using the chemiluminescent substrate (CSPD) from Phospha-Light kit (Tropix) following the manufacturer's instructions. Light signal output expressed as relative light units (RLU) was measured 45 minutes after the addition of the CSPD substrate using a luminometer (Envision 2102 Multilabel reader, Perkin Elmer). The neutralisation titre was defined as the reciprocal of sera dilution required to inhibit SEAP expression by 50% compared to the SEAP expression of virus infection alone. The lowest serum dilution tested was 1:18, therefore, a neutralisation titre of <1:18 is considered negative.

**T cell ELISpot assays**

Ex vivo (18 hour stimulation) IFNγ ELISpot assays were performed using freshly isolated PBMC as previously described [3]. Peptides were 15mers in length overlapping by 11 amino acids. Pools of peptides were prepared at 2.5 μg/ml according to the structural elements of the glycoprotein. These consisted of a pool of peptides corresponding to the signal peptide (SP: aa 1-32), seven pools comprised of peptides from the glycoprotein chain 1 (GP1: aa 33-501) and two pools comprised of peptides from the glycoprotein chain 2 (GP2: aa 502-676). Responses were averaged across triplicate wells, and then responses in unstimulated (negative control) wells were subtracted. The peptide pools were then summed giving a total response to GP1 + 2 + SP.

**Intracellular cytokine staining and flow cytometry**

Intracellular cytokine staining was performed on freshly isolated PBMC at seven days after MVA boost as previously described [3]. Cells were stimulated with a pool containing all 187 peptides spanning the glycoprotein at 2.5μg/ml. Samples were analysed on an LSR II cytometer (Becton Dickinson, Oxford, UK). Stimulation, cytokine staining and flow cytometry for EBL06 was conducted in the same way using fresh PBMC in Senegal and both data sets were analysed together using FlowJo v9.8.1 (Treestar Inc., Ashland, Oregon, USA). A hierarchical gating strategy was used. Responses to peptide were determined after subtraction of the response in the unstimulated control for each sample. For analyses of multiple cytokine function, all samples had >21,000 CD4^+^ or CD8^+^ T cells in the parent population. Samples were excluded from analysis if a response to the positive control of greater than 1% cytokine positive CD4^+^ or CD8^+^ T cells could not be detected. The lower limit of detection for the assay was 0.005% and a positive response was greater than two times the medium control for the corresponding sample.

1. Ewer K, Rampling T, Venkatraman N, et al. A Monovalent Chimpanzee Adenovirus Ebola Vaccine Boosted with MVA. The New England journal of medicine **2016**; 374:1635-46.

2. Aste-Amezaga M, Bett AJ, Wang F, et al. Quantitative adenovirus neutralization assays based on the secreted alkaline phosphatase reporter gene: application in epidemiologic studies and in the design of adenovector vaccines. Hum Gene Ther **2004**; 15:293-304.

3. Ewer K, Rampling T, Venkatraman N, et al. A Monovalent Chimpanzee Adenovirus Ebola Vaccine Boosted with MVA. N Engl J Med **2016**; 374:1635-46.
